# A survey of the prevalence of modifiable health risk behaviours among carers of people with a mental illness

**DOI:** 10.1186/s12889-019-7577-4

**Published:** 2019-09-09

**Authors:** Jacqueline M. Bailey, Tim W. Regan, Kate M. Bartlem, John H. Wiggers, Paula M. Wye, Jenny A. Bowman

**Affiliations:** 1 School of Psychology, Faculty of Science and Information Technology, University of Newcastle, University Drive, Callaghan, NSW 2308 Australia; 2 Hunter Medical Research Institute, Clinical Research Centre, Lot 1 Kookaburra Circuit, New Lambton Heights, NSW 2305 Australia; 3 Population Health, Hunter New England Local Health District, Booth Building, Wallsend Health Services, Longworth Avenue, Wallsend, NSW 2287 Australia; 4 School of Medicine and Public Health, Faculty of Health and Medicine, University of Newcastle, University Drive, Callaghan, NSW 2308 Australia

**Keywords:** Mental health carers, Smoking, Alcohol, Physical activity, Health risk behaviours

## Abstract

**Background:**

Family carers provide significant support to people with a mental illness; yet may experience poor mental and physical health themselves. Among limited research addressing the physical health of carers, studies of carers of people with dementia and young people with psychosis suggest increased risk of chronic diseases in conjunction with higher levels of potentially modifiable lifestyle risk behaviours. This exploratory study, conducted with carers of people with various mental illnesses, aimed to determine: carer prevalence of health risk behaviours (inadequate fruit and vegetable consumption, inadequate physical activity, harmful alcohol consumption, and tobacco smoking); interest in changing ‘at risk’ behaviours; and potential associations of socio-demographic characteristics with risk status and interest in change.

**Methods:**

A cross-sectional survey was conducted among family carers of people with a mental illness (*N* = 144) residing in New South Wales, Australia. Analyses explored risk behaviour prevalence and interest in change, and associations with socio-demographic variables.

**Results:**

Inadequate fruit and vegetable consumption was most prevalent (74.8%), followed by engaging in inadequate amounts of physical activity (57.6%); harmful alcohol consumption (36.3%) and smoking (11.8%). The majority of carers were interested in improving ‘at risk’ behaviours (56.3–89.2%), with the exception of alcohol consumption (41.5%). Previously or never married participants were more likely to consume inadequate amounts of fruits and/or vegetables compared to those married or cohabiting (Odds Ratio [OR]: 4.1, 95% Confidence Interval [CI]: 1.3–12.9, *p* = .02). Carers in the workforce were more likely to be engaging in inadequate physical activity (OR: 2.6, 95% CI: 1.2–5.7, *p* =  .02); and male participants were more likely to engage in harmful alcohol consumption (OR: 2.9, 95% CI: 1.1–7.9, *p* = .03). Working carers were approximately five times more likely to report interest in improving their alcohol consumption (OR: 5.1, 95% CI: 1.3–20.5, *p* = .02) compared to those not currently in the workforce.

**Conclusions:**

Results suggest high engagement in health risk behaviours among carers of people with a mental illness, particularly with regards to harmful alcohol consumption. Findings suggest a need to develop and implement chronic disease prevention strategies. Further research with larger representative samples is needed to confirm findings.

**Electronic supplementary material:**

The online version of this article (10.1186/s12889-019-7577-4) contains supplementary material, which is available to authorized users.

## Introduction

It is estimated that approximately half a million (476,000) Australians act as informal family carers for a family member or friend with a mental illness [1], with numbers in the United Kingdom (UK) and United States (US) estimated at 5 million and 43.5 million respectively [2, 3]. The significant role such carers play in providing unpaid emotional, social, financial and health care support for people with a mental illness is increasingly recognised internationally [4–6], as is the need to provide appropriate support to sustain their caregiving capacity [4, 7–9].

Internationally, family carers have been found to experience poor physical and mental health, with the caring role a direct contributor to such health deficits [2, 4, 7–10]. A UK survey of 1007 family carers for instance - among whom 26% cared for someone with a mental health problem - found that almost one third (30%) rated their own health as only fair or poor. Approximately half (49%) said they had a longstanding physical complaint of some description, while one fifth (21%) reported they had to restrict their activities in the 2 weeks before interview because of a health problem. Almost one quarter (24%) reported their caring responsibilities had affected their health in one or more ways at least a little of the time-most often worry, depression and tiredness; with 14% indicating they had smoked, drank alcohol or used drugs more as a result of their caring responsibilities [10].

Research which has focused on carers of people with a mental health problem specifically similarly suggests a health burden associated with the caregiving role. In an Australian survey of 756 carers of people with a mental illness for instance, 71% of participants perceived their health deteriorated as a direct result of assuming their caring role [11]. A nationally representative Australian household survey (*n* = 8841) reported that, in comparison to non-carers, family carers of a relative with any mental health condition (*n* = 1309) were more likely to experience both a mental health condition at some point in their lifetime as well as recently elevated levels of distress [12]. Further, 31% of carers reported that they had been unable to provide care for between 1 and 7 days in the last month due to their own health problems; with 13% unable to care for greater than 7 days [12]. Such findings suggest a need to develop strategies to improve the physical health of carers for their own benefit, and to ensure their continued capacity to provide informal care to people with a mental illness.

Much of the research examining the health consequences of caring for someone with a mental illness has focused on the impact on carers’ mental well-being with little previous research exploring the impact on physical health. A related body of literature- carers of people with neurodegenerative disorders such as dementia- however, has drawn attention to the poor physical health carers are also likely to experience. A recent survey in Japan for instance, of family carers of people with Alzheimer’s disease (*n* = 1302) and non-caregivers (*n* = 53,758), found that carers had relatively poorer health and greater comorbid risk than non-caregivers across a majority of health outcome measures, including: greater depression, more frequent insomnia, anxiety, hypertension, pain, and diabetes, higher rates of smoking and alcohol consumption; and poorer health-related quality of life scores, with respect to both mental and physical health domains [13]. Additionally, US studies of carers of people with Alzheimer’s disease have reported higher coronary heart disease risk and higher blood pressure relative to non-carer controls [14], and higher smoking rates where 40% of carers were current smokers [15]; compared to 15% of adults among the general population [16].

The studies above suggest a high prevalence of potentially modifiable health risk behaviours among dementia carers, with implications for an increased risk of preventable chronic diseases such as cardiovascular disease, cancer and diabetes [17, 18]. Among the general population, the four leading modifiable chronic disease risk factors are: inadequate fruit and vegetable consumption, inadequate physical activity, harmful alcohol consumption, and tobacco smoking [18]. Fruit and vegetable consumption, as opposed to nutrition more broadly, is implicated as one of the four leading modifiable chronic disease risk behaviours due to the association with the development of a number of chronic diseases including: ischemic heart disease; stroke; diabetes mellitus; and cancer [19–24]. Limited research to date has explored the prevalence of such health risk behaviours among carers of people with a broad range of mental illness. In Australia, a recent study of 42 carers of young people with psychosis suggested that this sub-group of carers may also be at high risk for chronic disease and especially so for the development of type 2 diabetes, with 79% of carers overweight and one-third with hypertension, and 24% being daily smokers [25]. Despite the potential implications however for an increased chronic disease risk for carers and the possible need for targeted preventive efforts, there has to date been a lack of similar research among carers of people with a broader range of mental illness to identify either the prevalence of health risk behaviours or carers’ interest in risk behaviour change. Given existing knowledge gaps, the present exploratory study was undertaken with a sample of carers of people with mental illness and aimed to:

1. Examine the prevalence of health risk behaviours (inadequate fruit and vegetable consumption, inadequate physical activity, harmful alcohol consumption, and tobacco smoking)
2. Assess interest in improving health risk behaviours
3. Explore possible associations of sociodemographic characteristics with health risk behaviour status, and interest in changing risk behaviours.

## Method

### Design and setting

A cross-sectional survey was distributed to carers of people with a mental illness in one non-metropolitan region of New South Wales (NSW), Australia from July to November 2013. The study was approved by the Hunter New England Human Research Ethics Committee (No. 13/06/19/5.11) and was registered with the University of Newcastle’s Human Research Ethics Committee (No. H-2013-0343). The STrengthening the Reporting of OBservational studies in Epidemiology (STROBE) checklist informed the reporting of findings (refer to Additional file 2 for completed checklist).

### Participants and recruitment

Potential participants were members of a non-government carer support organisation located in the study region that provided support services, training, and education to carers of people with a mental illness at no cost [26]. The focus of the service was to assist carers in their role of supporting individuals with a mental illness in the home environment and in navigating the mental health system/services. Inclusion criteria on the information statement required potential participants to be: over the age of 18 years, self-identified as a carer for anyone with a mental illness who was also over 18 years of age, and affiliated with the carer support organisation.

Carer members of the support organisation who previously indicated consent to receive invitations to participate in research were invited to participate in the study. The organisation posted an invitation to participate in the study, information statement, the survey instrument and reply-paid envelope, and instructions and a web link to complete the survey online if preferred to all such listed members. After 1 month, participants who had not responded were mailed a letter reminding them of their invitation to complete the survey. Additional participants were approached by members of the research team through attendance at carer support group meetings organised by or affiliated with the carer organisation.

### Data collection procedures

Socio-demographic and risk behaviour items were adapted from previous published research among people with a mental illness and clients of community health services conducted in Australia [27–30]. Items detailing the caring relationship and interest in changing risk behaviours were developed for the current study in the absence of pre-existing validated measures.

### Measures

#### Socio-demographic characteristics

Six items asked: carer age, gender, employment status, marital status, highest level of education achieved, and identification as an Aboriginal or Torres Strait Islander person. Participants were also asked: their postcode of residence to determine geographic remoteness [31] (major cities, inner regional, outer regional) and socio economic index using the Index of Relative Socio-economic Advantage and Disadvantage [32] (disadvantaged, average/advantaged) of the area they resided in; how many years they had been in a caring role for the person they cared for (years: less than 1 year, 1 to 2, 3 to 10, 11 to 20, more than 20); if they lived in the same residence (yes, no, sometimes); and what their relationship was to that person (parent, partner, child, sibling, neighbour, friend, other). Participants answered four items about the person with a mental illness for whom they provided care: age, gender, employment status, and primary psychiatric diagnosis (schizophrenia, depression, anxiety disorder, panic disorder, bipolar disorder, post-traumatic stress disorder, eating disorder, personality disorder, dementia, unsure, other).

#### Health risk behaviour status

Risk behaviours were assessed with five to seven questions, depending on responses given. Items queried current fruit and vegetable intake, physical activity levels, alcohol consumption and tobacco smoking status and were adopted from previous research [27]. Participants reported: the number of serves of vegetables consumed each day (0, 1, 2, 3, 4, 5 or more, unsure); number of serves of fruit consumed each day (0, 1, 2 or more, unsure); days a week they engaged in at least 30 min of physical activity (0, 1, 2, 3, 4, 5, 6, 7, unsure, can’t do physical activity for health or treatment reasons); if they were a current smoker (yes- daily, yes- at least once a week, yes- less than once a week, no- trying to quit, no-quit longer than 4 months ago, no- never smoked); how often they have a drink containing alcohol (never- not drinking alcohol, monthly or less, 2 to 4 times a month, 2 to 3 times a week, 4 or more times a week, unsure); number of standard drinks consumed on a typical drinking day (1 to 2, 3 to 4, 5 to 6, 7 to 9, 10 or more, unsure); frequency of consuming four or more standard drinks on one occasion (never, less than monthly, monthly, weekly, daily or almost daily, unsure). Risk status for each behaviour was subsequently determined based on the Australian national guidelines at the time of the study, where risk was defined as: consuming less than five serves of vegetables or two serves of fruit each day [33]; engaging in less than 30 min of physical activity on at least 5 days per week [34]; consuming more than two standard alcoholic drinks on a regular day (chronic harmful consumption) or more than four standard drinks on any one occasion (binge consumption) [35]; and any tobacco consumption [36]. Such definitions of risk have also been used in research reporting the prevalence of such risks among people with a mental illness [28, 29], and clients of community health services [30].

#### Interest in improving health risk behaviours

Participants were asked one item for each risk behaviour: if in the last year they had an interest in improving the behaviour (increasing fruit and vegetable consumption, increasing physical activity, decreasing alcohol consumption, quitting smoking) (yes, no, unsure). A ‘not applicable’ option was available for instances where the participant was not a smoker or did not drink alcohol.

### Data analysis

SPSS version 23 was used to analyse the data [37]. All responses to socio-demographic, and risk behaviour status items were collapsed into two categories, except responses to age, education, geographic remoteness and psychiatric diagnosis variables which were collapsed to three categories (Tables 1 and 2). Highest education level (less than 4 years high school completed, 4 years high school completed, more than 4 years high school completed) was categorised based on Australian standards where 4 years of high school is the minimum required level of schooling. Carer age (18–54, 55–74, 75 and over) and person with a mental illness age (18–34, 35–54, 55 and over) categories were collapsed to three categories in order to conduct more meaningful analysis by ensuring sufficient numbers were contained in each category. Not applicable responses for items examining interest in change were excluded from analysis.

**Table 1:** Socio-demographic characteristics

Characteristic	Carer *n*(%)	Person with a mental illness *n*(%)
Age (Years) ^a^		
18–34	6 (4.2)	58 (40.3)
35–54	29 (20.4)	67 (46.5)
55–74	92 (64.8)	18 (12.5)
75 and over	15 (10.6)	1 (0.7)
Gender ^a^		
Male	27 (19.0)	96 (66.7)
Female	115 (81.0)	48 (33.3)
Employment status		
In the workforce ^b^	45 (31.9)	28 (20.3)
Not in the workforce	96 (68.1)	110 (79.7)
Ethnicity ^c^		
Aboriginal and/or Torres Strait Islander origin	5 (3.6)	
Neither Aboriginal nor Torres Strait Islander	135 (96.4)	
Marital Status ^d^		
Married/ living together in a relationship	105 (73.4)	
Previously married/ never married	38 (26.6)	
Highest Education Level ^e^		
Less than 4 years high school completed	28 (19.6)	
4 years high school completed	30 (21.0)	
More than 4 years high school completed	85 (59.4)	
Index of Relative Socio-economic Advantage and Disadvantage ^a^		
Lowest tertile (Disadvantaged)	78 (54.9)	
Middle/highest tertile (average/advantaged)	64 (45.1)	
Geographic remoteness ^a g^		
Major cities	44 (31.0)	
Inner regional	77 (54.2)	
Outer regional	21 (14.8)	
Years spent caring for the person with mental illness ^a^		
20 years or less	100 (70.4)	
More than 20 years	42 (29.6)	
Carer and person with mental illness living in the same residence		
Yes ^f^	75 (52.4)	
Carer relationship to person with mental illness ^f^		
Parent	88 (61.5)	
Other relation	55 (38.5)	
Psychiatric diagnosis		
Schizophrenia		56 (39.1)
Bipolar disorder		31 (21.8)
Other disorder		56 (39.1)

^a^ 2 missing carer responses

^b^ 3 missing carer responses, 6 missing person with a mental illness responses

^c^ 4 missing carer responses

^d^ 1 missing carer response

^e^ 1 missing carer response

^f^ 1 missing carer response

^g^ 0 participants resided in ‘remote’ or ‘very remote’ areas

**Table 2:** Prevalence of chronic disease risk behaviours and interest in change

Carer Characteristic	*n*(%)
Nutrition	
At risk for inadequate fruit consumption	49 (34.0)
At risk for inadequate vegetable consumption ^a^	98 (68.5)
At risk for inadequate fruit or vegetable consumption ^a^	107 (74.8)
Those ‘at risk’ for inadequate fruit or vegetable consumption interested in change ^b^	62 (63.9)
Physical activity	
At risk for inadequate physical activity ^a^	76 (57.6)
Those ‘at risk’ interested in change ^c^	66 (89.2)
Alcohol consumption	
At risk for binge alcohol consumption ^b^	46 (34.1)
At risk for chronic harmful alcohol consumption ^b^	26 (19.3)
At risk for chronic and/or binge alcohol consumption ^b^	49 (36.3)
Those ‘at risk’ for chronic and/or binge consumption interested in change ^d^	17 (41.5)
Smoking	
At risk for smoking	17 (11.8)
Those ‘at risk’ interested in change ^a^	9 (56.3)

^a^ 1 missing response

^b^ 9 missing responses

^c^ 2 missing responses

^d^ 8 missing responses

Descriptive statistics were used to summarise the socio-demographic characteristics of carers and people with a mental illness, carer risk behaviour status, and interest in behaviour change (Tables 1 and 2). Analysis of interest in change was restricted to carers who were classed as ‘at risk’ for each respective behaviour.

Chi-square analyses were used to examine possible associations between all sociodemographic variables (independent variable) with carer risk behaviour status (at risk, not at risk) (dependent variable) and carer interest in improving risk behaviours (yes, no/unsure) (dependent variable). Independent variables associated at *p* < .25 were subsequently entered into a multivariate logistic regression model to examine the association (*p* < .05) to risk behaviour status (one model per behaviour, where possible) and interest in improving risk behaviours (one model per behaviour, where possible) (eight models total, where possible). The use of this *p*-value (*p* < .25) is recommended as variables which may be of clinical relevance may be discounted using the more traditional levels of *p* < .05 [38]. Missing responses were excluded from analysis.

Independent variables entered into the regression model for fruit and vegetable consumption risk status were: carer gender, carer employment status, carer marital status, and carer relationship to the person with a mental illness. Variables entered into the regression model for physical activity risk status were: carer age, carer employment status, and years caring for the person with a mental illness. Variables entered into the regression model for alcohol consumption risk status were: carer age, carer gender, and carer highest education level. Variables entered into the regression model for interest in changing alcohol consumption were: carer employment status, and Index of Relative Socio-economic Advantage and Disadvantage (Additional file 1: Table S1, Table 3).

**Table 3:** Variables associated with chronic disease risk behaviour status and interest in changing ‘at risk’ behaviours

Variable*	OR	95% CI	*p*
Fruit and Vegetable Consumption Risk Status ^(*n* = 137)a^			
Carer previously married/never married	4.12	1.31–12.94	.02
Carer married or cohabiting		Reference category	
Physical Activity Risk Status ^(*n* = 127)b^			
Carer in the workforce	2.58	1.17–5.70	.02
Carer not in the workforce		Reference category	
Alcohol Consumption Risk Status ^(*n* = 133)c^			
Male carer	2.98	1.12–7.97	.03
Female carer		Reference category	
Interest in Changing Alcohol Consumption ^(*n* = 40)d^			
Carer in the workforce	5.14	1.29–20.52	.02
Carer not in the workforce		Reference category	

*OR* Odds Ratio, *CI* Confidence Interval

* No significant associations were found for smoking risk status, interest in changing fruit and vegetable consumption, and smoking. Carer age was found to be significantly associated with interest in improving physical activity, however the small number of included negative event cases where carers were not interested in improving physical activity (8/63) contributed to large 95% CI; thus the authors had low confidence in the accuracy of the model and results are not reported

Variables entered into regression models:

^a^ Carer gender, carer employment status, carer marital status, carer relationship to person with a mental illness

^b^ Carer age, carer employment status, years caring for person with a mental illness

^c^ Carer age, carer gender, carer highest education level

^d^ Carer employment status, Index of Relative Socio-economic Advantage and Disadvantage

## Results

### Sample characteristics

Of 383 invitations to participate in the study (327 by post, 56 by support group invitation), 144 carers consented to participate (37.6%). The majority of non-consenters (227, 69.5%), did not respond to the posted invitation, 12 were ineligible (3.7%) after making contact with the research team (person under 18 years; no longer in a caring role). A total of 97 participants (29.6% of 327 invited) completed the posted survey, 46 (82.1% of 56 invited) completed the survey during a carer support group, and 1 participant completed the survey online. Participants who completed the survey during a support group were more likely to be 75 years or older (21.7% vs 7.1%, *p* = .005) and live in a major city (57.8% vs 18.6%, *p* < .001) than those who completed the survey by post.

The majority of participants were female (81.0%), over the age of 54 (75.7%, mean = 59.5, standard deviation [SD] = 11.5), the parent of the person they cared for (61.5%), and living in the same residence with the person they cared for (52.4%). The majority of participants were caring for a person who was male (66.7%); not currently in the workforce (79.7%); and between the ages of 18 and 54 (86.8%, mean = 40.5, SD = 13.6). Thirty-nine percent were caring for a person with a diagnosis of schizophrenia (Table 1).

### Health risk behaviour status

The health risk behaviour for which most participants were at risk was inadequate fruit and vegetable consumption where 74.8% reported consuming inadequate serves of fruit and/or vegetables per day. Approximately two thirds (57.6%) of participants were engaging in inadequate amounts of physical activity. Approximately one third were at risk for combined harmful alcohol consumption; 19.3% for chronic harmful alcohol consumption; and 34.1% for binge alcohol consumption. A smaller proportion (11.8%) of the sample were current smokers (Table 2). Approximately two-thirds of participants were at risk for multiple behaviours: with 4.8% at risk for all four; 26.4% at risk for three; and 31.2% at risk for two. One quarter were at risk for a single behaviour only (25.6%), and 12% for none.

### Interest in improving health risk behaviours

Of the participants ‘at risk’ for each behaviour, the majority (56.3–89.2%) were interested in improving that behaviour. The exception was alcohol consumption, with 41.5% interested in improving their chronic harmful and/or binge alcohol consumption (Table 2).

### Sociodemographic associations with health risk behaviour status and interest in improving ‘at risk’ behaviours

#### Health risk behaviour status

Previously or never married participants were more likely to consume inadequate amounts of fruits and/or vegetables compared to those married or cohabiting (Odds Ratio [OR]: 4.1, 95% Confidence Interval [CI]: 1.3–12.9, *p* = .02). Carers who were currently in the workforce were more likely to be engaging in inadequate physical activity compared to those not in the workforce (OR: 2.6, 95% CI: 1.2–5.7, *p* = .02); and male participants were more likely to engage in harmful alcohol consumption as opposed to female participants (OR: 2.9, 95% CI: 1.1–7.9, *p* = .03). No variables were found to be significantly associated with tobacco smoking.

#### Interest in improving health risk behaviours

Participants currently in the workforce were approximately five times more likely to report interest in improving their alcohol consumption (OR: 5.1, 95% CI: 1.3–20.5, *p* = .02) compared to those not currently in the workforce. No variables were found to be significantly associated with interest in improving fruit and vegetable consumption. Additionally, no variables were found to be significantly associated with interest in improving smoking behaviours, however the small sample size (*n* = 15) limits the confidence in this finding. Furthermore, carer age was found to be significantly associated with interest in improving physical activity, however the small number of negative event cases (where carers were not interested in improving physical activity, *n* = 8/63) contributed to large 95% CI; thus the authors had low confidence in the accuracy of the model and results are not reported (Table 3).

## Discussion

This is the first study to explore the prevalence of four major health risk behaviours and interest in improving them among carers of people with a mental illness. The prevalence of risk identified across the behaviours examined was substantial, being greatest for inadequate fruit and/or vegetable consumption (74.8%), followed by inadequate physical activity (57.6%), harmful chronic and/or binge alcohol consumption (36.3%) and smoking (11.8%). Carers who were previously or never married, in the workforce, and male, were more likely to be at risk for insufficient fruit and vegetable consumption, physical activity and harmful alcohol consumption respectively. Most carers who reported being smokers or being at risk of inadequate physical activity or fruit and vegetable consumption were interested in improving that behaviour, whereas less than half of carers at risk of harmful alcohol consumption expressed interest in doing so. Participants who were currently in the workforce were more likely to express interest in improving their alcohol consumption.

Results suggest carers of people with a mental illness may not experience a higher prevalence of chronic disease risk behaviours compared to the general adult population, with the exception of harmful alcohol consumption. Similarly high levels of risk for inadequate fruit and vegetable consumption as identified among the present carer sample have also been found among the general Australian population: a 2011–12 survey of adults (aged 18 years and above) indicating over 70% consumed inadequate fruit and over 90% consumed inadequate vegetables [39]. Also reflective of similar levels of risk to those identified among the present carer sample, data from recent surveys of the general adult population indicate just over half (52%) to engage in inadequate physical activity [40] and 12% to be smokers [41]. Such comparisons with general population figures however must be made cautiously, given the skewed demographic profile of carers in the present study (predominantly female and aged over 54 years). However, when the alcohol consumption of the present carer sample is compared to a general Australian population sample of similar age profile (55–64 years), while the prevalence of harmful chronic alcohol consumption is closely comparable (19.3% vs. 20%), the prevalence of harmful binge alcohol consumption among the present carer sample (34.1%) is notably higher than among the general population sample (21%) [41].

It is difficult to directly compare the prevalence of risk behaviours identified in the present carer sample to the limited previous research involving carers for a number of reasons including the different countries across which such research has been conducted and the differences in characteristics of carers including the nature of the mental health conditions of those being cared for [13, 15, 25, 42]. In comparison to a recent Australian study however, involving 42 carers of young people with psychosis and where the same approach to the classification of risks was used [25], carers in the present study had a lower prevalence of smoking (11.8% vs 24%) and higher likelihood of engaging in inadequate physical activity (57.6% vs 31%).

Comparison of the findings of this study with respect to factors associated with chronic disease risk among carers of people with a mental health condition, is similarly fundamentally limited by an absence of previous carer research. One recent Australian study examined health and well-being more broadly among carers of elderly family members with a disability, and found a greater decline in carer health and wellbeing for those in the workforce as compared to those who were not, a finding which might be seen to bear some relation to that in the present study where carers in the workforce were more likely to engage in inadequate physical activity than those not in the workforce [43].

A number of findings in the present study relating to associations with risk behaviours however do appear to reflect findings among general population samples. Among the Australian general population for instance, males are more likely than females to consume alcohol harmfully [44]. Similarly, general population research conducted in the US [45, 46] and UK [47, 48] suggests poorer nutrition among single or previously married people compared to married people.

Finally it is difficult to explain the only association with interest in change found in the present study. It might be speculated that, given the significant impact harmful alcohol use can have on work-related factors such as absenteeism [49], that those carers in the workforce may have experienced/noticed such negative impacts of excessive alcohol consumption on their work attendance or performance and so have been motived to change. Future research however would be required to examine such possible explanations.

A need exists for further research to confirm or otherwise the generalisability of these findings. If confirmed, the findings suggest that carers generally, and those caring for people with a mental illness represent a key group at risk of chronic disease and may require risk reduction programs particularly tailored to their caring roles/context [7]. Some research suggests that carers may require tailored support to address their risk behaviours, as their capacity to do so might be negatively impacted by factors such as: a personal reduction in the prioritisation of their health and wellbeing when compared to the people they care for [50, 51]; time, transport and financial constraints on seeking professional support to address risk behaviours [7]; or a neglect of their own physical wellbeing by health professionals when attending appointments with the person they care for [51]. Such support may also need to take account of the fluctuation and unpredictable nature of the caring input that might be required at a particular point in time [50, 52, 53]. Therefore, given the current findings of interest in changing at risk behaviours, and the potential for carers to experience a reduced capacity to address their health risk behaviours, interventions tailored to the circumstances and needs of carers are required. No evidence has been reported that describes the effectiveness of such interventions. Further research is therefore required to explore interventions to improve and support carer health risk behaviours.

A range of services are available to carers of people with a mental illness in Australia [26, 54–56]. Support services are free of charge and most offer individual counselling and/or group meetings aimed at providing emotional, educational, and practical (e.g. attending mental health service care planning meetings with carers) support for carers of people with a mental illness [26, 54, 55]. Such support services are often provided face to face and as such, rural and remote carers may experience disadvantage in access. However, online forums moderated by mental health professionals are also available nationwide for carers to provide and receive support from other carers [56]. Such support services may represent appropriate contexts in which interventions to improve carer health risk behaviours may be delivered. It is unknown if such services currently provide support for health risk behaviours; a potential avenue for further investigation.

The need to support all domains of carer well-being, including their physical health, has also been recognised in a number of national and international policy documents and surveys [4, 7, 8, 57]. The need to identify evidence-based strategies to support carers and to assist them in maintaining their health risk behaviours is further reinforced by recent estimates that the cost to replace informal care with paid care in Australia would exceed AUD$60 billion [58]. Further, data from the Australian Bureau of Statistics suggests that disability care needs for people with mental and behavioural disorders will more than double by the year 2031 [59].

### Limitations

The results of this research should be considered in the context of the following limitations. This study had a small sample (*N* = 144), with a low response rate (< 38%), drawn from members of a carer support organisation within one regional health district in Australia. All participating carers were affiliated with a carer support organisation, the majority experienced socio-economic disadvantage and resided in regional areas. As such the experiences of these carers may not be representative of all carers of people with a mental illness, suggesting a need for caution when interpreting findings; although the socio-economic and geographic characteristics of participants are largely consistent with characteristics of carers in Australia [60]. The self-report data may be prone to recall and social desirability biases [61]; perhaps most likely resulting in an under-estimation of engagement in risk behaviours and over-estimation of perceived interest in behaviour change. Some evidence does suggest however that older adults’ recall of their health behaviours is reliable [62].

## Conclusion

This study adds to the limited research regarding the prevalence of chronic disease risk behaviours among carers of people with a mental illness, and carers’ interest in improving risk behaviours and highlights the need for further research with larger representative samples. Findings suggest that carers may experience similar prevalence rates of health risk behaviours to the general population, requiring attention to reduce risks. Additionally, carers may experience a higher prevalence of harmful alcohol consumption, suggesting a potential focus for behaviour change strategies among this population group. The findings are relevant to clinicians and researchers who are looking to develop chronic disease prevention strategies to support and improve the health of carers of people with a mental illness.

## Additional files

Additional file 1: **Table S1** Chi-square results for variables associated with risk behaviour status and interest in changing ‘at risk’ behaviours. (DOCX 20 kb)

Additional file 2: STROBE Statement. Checklist of items that should be included in reports of cross-sectional studies. (DOC 87 kb)

## Data Availability

The dataset used and analysed during the current study is available from the corresponding author on reasonable request.

## Abbreviations

AUD: Australian Dollar
CI: Confidence Interval
NSW: New South Wales
OR: Odds Ratio
SD: Standard Deviation
STROBE: Strengthening the Reporting of Observational studies in Epidemiology
UK: United Kingdom
US: United States
