# Supplementary material for: A survey of the prevalence of modifiable health risk behaviours among carers of people with a mental illness
Source: BMC Public Health. 2019 Sep 9;19:1240. doi: 10.1186/s12889-019-7577-4 (PMC6734289; doi:10.1186/s12889-019-7577-4)
Supplement: Supplementary file 1 — Table S1 Chi-square results for variables associated with risk behaviour status and interest in changing ‘at risk’ behaviours. (DOCX 20 kb) [file 12889_2019_7577_MOESM1_ESM.docx]

**Supplementary Table 1:** Chi-square results for variables associated with risk behaviour status and interest in changing ‘at risk’ behaviours

| Predictor | N^a^ | $\chi$^2^ | *df* | *p* |
| --- | --- | --- | --- | --- |
| Fruit and Vegetable Consumption Risk Status |  |  |  |  |
| Carer age^b^ |  |  |  |  |
| Person with a mental illness age^b^ |  |  |  |  |
| Carer gender | 141 | 2.017 | 1 | .220* |
| Person with a mental illness gender | 143 | .001 | 1 | 1.000 |
| Carer employment status | 140 | 3.155 | 1 | .095* |
| Person with a mental illness employment status | 137 | .387 | 1 | .621 |
| Carer marital status | 142 | 6.026 | 1 | .016* |
| Carer highest education level | 142 | 1.658 | 2 | .458 |
| Carer ethnicity^b^ |  |  |  |  |
| Geographic remoteness | 141 | 1.870 | 2 | .396 |
| Index of Relative Socio-economic Advantage and Disadvantage | 141 | .178 | 1 | .702 |
| Person with a mental illness psychiatric diagnosis | 143 | 1.326 | 2 | .546 |
| Years caring for person with a mental illness | 141 | 1.160 | 1 | .294 |
| Residing in the same residence | 142 | .462 | 1 | .564 |
| Carer relationship to person with mental illness | 142 | 2.750 | 1 | .167* |
|  |  |  |  |  |
| Physical Activity Risk Status |  |  |  |  |
| Carer age | 132 | 5.899 | 2 | .053* |
| Person with a mental illness age | 132 | .821 | 2 | .678 |
| Carer gender | 130 | .635 | 1 | .508 |
| Person with a mental illness gender | 132 | .032 | 1 | 1.000 |
| Carer employment status | 129 | 6.311 | 1 | .014* |
| Person with a mental illness employment status | 126 | .522 | 1 | .514 |
| Carer marital status | 131 | .613 | 1 | .550 |
| Carer highest education level | 131 | 2.064 | 2 | .361 |
| Carer ethnicity^b^ |  |  |  |  |
| Geographic remoteness | 131 | 2.571 | 2 | .270 |
| Index of Relative Socio-economic Advantage and Disadvantage | 131 | .283 | 1 | .602 |
| Person with a mental illness psychiatric diagnosis | 132 | 1.063 | 2 | .577 |
| Years caring for person with a mental illness | 130 | 2.051 | 1 | .171* |
| Residing in the same residence | 131 | .264 | 1 | .723 |
| Carer relationship to person with mental illness | 131 | .073 | 1 | .855 |
|  |  |  |  |  |
| Harmful Alcohol Consumption Risk Status |  |  |  |  |
| Carer age | 135 | 6.256 | 2 | .044* |
| Person with a mental illness age | 135 | .667 | 2 | .754 |
| Carer gender | 133 | 3.777 | 1 | .063* |
| Person with a mental illness gender | 135 | 1.025 | 1 | .346 |
| Carer employment status | 132 | .020 | 1 | 1.000 |
| Person with a mental illness employment status | 131 | .000 | 1 | 1.000 |
| Carer marital status | 134 | .222 | 1 | .690 |
| Carer highest education level | 134 | 7.705 | 2 | .019* |
| Carer ethnicity^b^ |  |  |  |  |
| Geographic remoteness | 134 | .394 | 2 | .850 |
| Index of Relative Socio-economic Advantage and Disadvantage | 134 | .024 | 1 | 1.000 |
| Person with a mental illness psychiatric diagnosis | 135 | .844 | 2 | .695 |
| Years caring for person with a mental illness | 134 | .072 | 1 | .847 |
| Residing in the same residence | 134 | .320 | 1 | .593 |
| Carer relationship to person with mental illness | 134 | .054 | 1 | .855 |
|  |  |  |  |  |
| Smoking Risk Status |  |  |  |  |
| Carer age^b^ |  |  |  |  |
| Person with a mental illness age^b^ |  |  |  |  |
| Carer gender^b^ |  |  |  |  |
| Person with a mental illness gender | 144 | 1.634 | 1 | .273 |
| Carer employment status | 141 | .763 | 1 | .412 |
| Person with a mental illness employment status^b^ |  |  |  |  |
| Carer marital status^b^ |  |  |  |  |
| Carer highest education level^b^ |  |  |  |  |
| Carer ethnicity^b^ |  |  |  |  |
| Geographic remoteness^b^ |  |  |  |  |
| Index of Relative Socio-economic Advantage and Disadvantage | 142 | 1.913 | 1 | .201* |
| Person with a mental illness psychiatric diagnosis^b^ |  |  |  |  |
| Years caring for person with a mental illness^b^ |  |  |  |  |
| Residing in the same residence | 143 | 5.993 | 1 | .017* |
| Carer relationship to person with mental illness | 143 | 6.983 | 1 | .013* |
|  |  |  |  |  |
| Interest in Changing Fruit and Vegetable Consumption | |  |  |  |
| Carer age^b^ |  |  |  |  |
| Person with a mental illness age^b^ |  |  |  |  |
| Carer gender | 95 | .611 | 1 | .457 |
| Person with a mental illness gender | 97 | 1.010 | 1 | .379 |
| Carer employment status | 95 | .438 | 1 | .651 |
| Person with a mental illness employment status | 96 | .117 | 1 | .788 |
| Carer marital status | 96 | .851 | 1 | .371 |
| Carer highest education level | 96 | .948 | 1 | .656 |
| Carer ethnicity^b^ |  |  |  |  |
| Geographic remoteness | 96 | 3.590 | 2 | .178* |
| Index of Relative Socio-economic Advantage and Disadvantage | 96 | .836 | 1 | .395 |
| Person with a mental illness psychiatric diagnosis | 97 | 2.233 | 2 | .365 |
| Years caring for person with a mental illness | 95 | 2.507 | 1 | .155* |
| Residing in the same residence | 96 | .958 | 1 | .395 |
| Carer relationship to person with mental illness | 96 | .851 | 1 | .389 |
|  |  |  |  |  |
| Interest in Changing Physical Activity |  |  |  |  |
| Carer age | 74 | 13.818 | 2 | .007* |
| Person with a mental illness age | 74 | 2.392 | 2 | .378 |
| Carer gender | 72 | .188 | 1 | 1.000 |
| Person with a mental illness gender | 74 | .055 | 1 | 1.000 |
| Carer employment status | 71 | 3.271 | 1 | .071* |
| Person with a mental illness employment status | 71 | .084 | 1 | 1.000 |
| Carer marital status | 73 | 1.327 | 1 | .421 |
| Carer highest education level | 73 | 2.581 | 2 | .332 |
| Carer ethnicity^b^ |  |  |  |  |
| Geographic remoteness | 73 | 1.174 | 2 | .606 |
| Index of Relative Socio-economic Advantage and Disadvantage | 73 | .393 | 1 | .712 |
| Person with a mental illness psychiatric diagnosis | 74 | 2.023 | 2 | .449 |
| Years caring for person with a mental illness | 74 | .681 | 1 | .409 |
| Residing in the same residence | 74 | .689 | 1 | .474 |
| Carer relationship to person with mental illness | 74 | .629 | 1 | .428 |
|  |  |  |  |  |
| Interest in Changing Alcohol Consumption |  |  |  |  |
| Carer age^b^ |  |  |  |  |
| Person with a mental illness age^b^ |  |  |  |  |
| Carer gender^b^ |  |  |  |  |
| Person with a mental illness gender | 41 | .057 | 1 | 1.000 |
| Carer employment status | 40 | 5.736 | 1 | .024* |
| Person with a mental illness employment status^b^ |  |  |  |  |
| Carer marital status^b^ |  |  |  |  |
| Carer highest education level^b^ |  |  |  |  |
| Carer ethnicity^b^ |  |  |  |  |
| Geographic remoteness^b^ |  |  |  |  |
| Index of Relative Socio-economic Advantage and Disadvantage | 41 | 3.347 | 1 | .112* |
| Person with a mental illness psychiatric diagnosis^b^ |  |  |  |  |
| Years caring for person with a mental illness^b^ |  |  |  |  |
| Residing in the same residence | 41 | .963 | 1 | .358 |
| Carer relationship to person with mental illness | 41 | .455 | 1 | .539 |
|  |  |  |  |  |
| Interest in Changing Smoking Status ^b^ |  |  |  |  |

^a^ Sample size varies due to missing responses

^b^ Inadequate sample size in response options to conduct analysis

* Variables entered into logistic regression model.
